# Supplementary material for: Correlations between Maternal and Fetal Outcomes in Pregnant Women with Kidney Failure
Source: J Clin Med. 2023 Jan 20;12(3):832. doi: 10.3390/jcm12030832 (PMC9917987; doi:10.3390/jcm12030832)
Supplement: Supplementary file 1 [file jcm-12-00832-s001.zip › jcm-2005095-supplementary.pdf]

Supplementary Materials:

Table S1. The maternal outcome in the patient who underwent dialysis.

		Value	Df	Asymp. Sig. (2-sided)	Exact Sig. (2-sided)	Exact Sig. (1-sided)
Total	Pearson Chi-Square	34.074[a]	1	0.000		
	Continuity Correction[b]	30.752	1	0.000		
	Likelihood Ratio	30.744	1	0.000		
	Fisher's Exact Test				0.000	0.000
	Linear-by-Linear Association	33.704	1	0.000		
	N of Valid Cases	92	1			
Acute renal failure	Pearson Chi-Square	6.388[a]	1	0.011		
	Continuity Correction[b]	4.793	1	0.029		
	Likelihood Ratio	6.531	1	0.011		
	Fisher's Exact Test				0.018	0.014
	Linear-by-Linear Association	6.220	1	0.013		
	N of Valid Cases	38	1			
Chronic renal disease	Pearson Chi-Square	5.333[a]	1	0.021		
	Continuity Correction[b]	3.000	1	0.083		
	Likelihood Ratio	5.822	1	0.016		
	Fisher's Exact Test				0.080	0.040
	Linear-by-Linear Association	4.889	1	0.027		
	N of Valid Cases	12				
